# Are There Inequities in Treatment of End-Stage Renal Disease in Sweden? A Longitudinal Register-Based Study on Socioeconomic Status-Related Access to Kidney Transplantation

**DOI:** 10.3390/ijerph14020119

**Published:** 2017-01-27

**Authors:** Ye Zhang, Johan Jarl, Ulf-G. Gerdtham

**Affiliations:** 1 Health Economics Unit, Department of Clinical Sciences, Lund University, 22381 Lund, Sweden; johan.jarl@med.lu.se (J.J.); ulf.gerdtham@med.lu.se (U.-G.G.); 2 Department of Economics, Lund University, 22363 Lund, Sweden

**Keywords:** education, inequities, income, kidney transplantation, socioeconomic factors

## Abstract

Socioeconomic status-related factors have been associated with access to kidney transplantation, yet few studies have investigated both individual income and education as determinates of access to kidney transplantation. Therefore, this study aims to explore the effects of both individual income and education on access to kidney transplantation, controlling for both medical and non-medical factors. We linked the Swedish Renal Register to national registers for a sample of adult patients who started Renal Replacement Therapy (RRT) in Sweden between 1 January 1995, and 31 December 2013. Using uni- and multivariate logistic models, we studied the association between pre-RRT income and education and likelihood of receiving kidney transplantation. For non-pre-emptive transplantation patients, we also used multivariate Cox proportional hazards regression analysis to assess the association between treatment and socioeconomic factors. Among the 16,215 patients in the sample, 27% had received kidney transplantation by the end of 2013. After adjusting for covariates, the highest income group had more than three times the chance of accessing kidney transplantation compared with patients in the lowest income group (odds ratio (OR): 3.22; 95% confidence interval (CI): 2.73–3.80). Patients with college education had more than three times higher chance of access to kidney transplantation compared with patients with mandatory education (OR: 3.18; 95% CI: 2.77–3.66). Neither living in the county of the transplantation center nor gender was shown to have any effect on the likelihood of receiving kidney transplantation. For non-pre-emptive transplantation patients, the results from Cox models were similar with what we got from logistic models. Sensitive analyses showed that results were not sensitive to different conditions. Overall, socioeconomic status-related inequities exist in access to kidney transplantation in Sweden. Additional studies are needed to explore the possible mechanisms and strategies to mitigate these inequities.

## 1. Introduction

Patients with End-Stage Renal Disease (ESRD) face many challenges, including increased burden of comorbidities, reduced life expectancy and impaired quality of life, compared to general population [1,2]. Renal Replacement Therapy (RRT) are lifesaving treatments which include dialysis and kidney transplantation. Kidney transplantation is generally regarded as the preferred treatment choice compared to dialysis as it not only extends patients’ life expectancy but also improves quality of life [3,4]. However, the available supply of kidneys cannot meet the demand, which leads to both long waiting lists and prolonged waiting times [5].

Previous studies have shown that inequities exist in access to kidney transplantation. Evidence, mainly from the USA, indicate that there is an association between kidney transplantation and race/ethnicity [6], gender [7], socio-economic status (SES) [8,9,10], marital status [11], and patient awareness [12]. The role of SES is complex because SES affects service along the pathway to transplantation [13], such as higher SES patients may have good communication with health care providers [14]. 

In order to explore the role of SES on access to kidney transplantation, the first thing is the definition of SES. Three classic defining indicators of SES are income, occupation, and education [14]. However, previous studies mostly used the ZIP code or residential postcode of patients to proxy SES measures [9,15,16,17]. Based on national data from Australia, Grace et al. [9] reported that patients with higher SES were more likely to receive pre-emptive and living-donor kidney transplantation, although no association could be found for deceased-donor kidney transplantation. Studies from the US have shown that high SES increased access to transplantation for both living [15] and deceased donor kidneys [15,16]. A study from the UK found that socioeconomic disadvantaged patients were less likely to be placed on the waiting list, although had equal chance of transplantation with socioeconomic advantaged patients once listed [17]. 

Two studies have investigated the effect of education on access to kidney transplantation in the US, however the effect of education was inconsistent. Schaeffiner et al. [14] showed that college graduates had three times greater chance to be waitlisted or receive kidney transplantation compared to patients without a high school degree. The international Dialysis Outcomes and Practice Patterns Study (DOPPS) controlled for income and education at the same time and found that only income was (positively) associated with access to kidney transplantation [18].

The studies above vary with regard to study design, patient selection, sample size, time period, statistical methodology, outcomes of interest, SES measures, and availability of potential confounders. Beyond this, the health care systems are also different across countries. For SES measures, previous studies generally only used one of the three classic defining indicators of SES, and (or) used the ZIP code or residential postcode data as the only SES indicator, which potentially lead to less accurate estimates of individual level SES [9,15,17]. Furthermore, earlier studies are also limited due to lack of major potential confounders (e.g., comorbidities [9,14]), only including a subsample of the population [8,9], or being single center studies [16].

Sweden is a country with universal health care system and has no waiting lists for ESRD care except for kidney transplantation, as assessment and allocation of kidneys are based on numerous factors [19]. Studying equity in access to kidney transplantation is especially of interest in the context of the health care system and the outspoken egalitarian welfare system in the Nordic countries. It has been a long tradition in these countries to reduce or even try to eradicate social inequality, both in health care where treatment should be given irrespective of socio-economic status and in the society at large. Unfortunately, the association between individual SES and access to register-based kidney transplantation has not previously been studied in Sweden or any other Nordic country. 

Therefore, the aim of this study is to assess the impact of SES on access to kidney transplantation using a Swedish population sample and register data. The main contributions of the present study are that we used longitudinal register-based individual SES measured as two of the main indicators of SES before start of RRT; individual disposable income and education. In addition, we also control extensively for both medical and non-medical factors.

## 2. Materials and Methods

### 2.1. Material

The Swedish Renal Register (SRR) [20] was linked to the Longitudinal Integration Database for Health Insurance and Labor Market Studies (LISA by Swedish acronym) [21] and the Register of the Total Population (RTB) from Statistics Sweden using the national personal identification numbers. The SRR is a national high quality register for patients undergoing RRT starting in 1991, with almost 100% coverage and a data reporting incidence of 95% [22]. The register includes rich information related to the patient, disease and treatment. 

The LISA data set combines information from several demographic and socioeconomic population registers [21], such as income, education, and employment status. These data are registered yearly for all older than 15 living in Sweden. Marital status and citizenship information comes from the RTB. 

### 2.2. Study Population

The study population was defined as all patients aged 18 years and older who started RRT between 1 January 1995, and 31 December 2013, as recorded in the SRR. During this period there have been no changes to the Swedish kidney allocation policy. Patients whose current treatment modality are unknown and patients who recovered or died within 91 days after start of RRT were excluded, in order to only include patients with chronic conditions. 

We lack information on counter-indication, i.e., we cannot identify patients that were never suitable for kidney transplantation. We therefore assumed that all patients starting renal replacement therapy were suitable for kidney transplantation.

(i) Study outcome and SES indicators

This study investigated the association between SES and access to kidney transplantation, in terms of probability of receiving kidney transplantation. Access to kidney transplantation was defined as receiving a first living- or deceased donor kidney transplant during the study period.

SES was measured in terms of income and education before the patient started RRT. Income was defined as the individual disposable income the year before the patient started RRT, derived from the household disposable income using consumption weights [23]. We adjusted disposable income to 2012 year’s price level using the Consumer Price Index (CPI) from Statistics Sweden [24]. The disposable income was divided into quintiles, with quintile 1 (0–99,998 SEK) being the most disadvantaged and quintile 5 (188,751–6,685,735 SEK) being the most advantaged. Education was divided into three categories based on the Swedish educational system: mandatory education (≤9 years), high school education (9–12 years), and college education (>12 years). 

(ii) Control (potentially confounding) variables

We identified several baseline demographic and clinical variables before the patient started RRT, in order to adjust for potential confounding based on prior studies. These factors included age at start of RRT [8,9], gender [8,9], year of first RRT [16], marital status [11], residence area (county) [9], citizenship [25], primary renal disease [8,9], and comorbidities [8,9]. Based on the distribution of transplantation events [26], age was divided into four age groups: 18–39, 40–49, 50–59, ≥60 years old. Year of first RRT was handled as a continuous covariate (per year). The marital status included married, single, divorced, and widow. In Sweden, there are four kidney transplantation centers, located in the four largest cities (Stockholm, Gothenburg, Malmo, and Uppsala). We created the binary variable home county to capture any potential advantage of living in the same administrative area that performed the transplantation and/or living close to the performing hospital. Citizenship was measured as Swedish or non-Swedish. The comorbidities in the Swedish renal register database had eight categories, but were re-categorized due to small samples in certain groups. Therefore, comorbidities were categorized as hypertension, diabetes mellitus, cancer (blood-, skin-, and other cancer) and heart disease (cerebrovascular-, peripheral vascular-, Ischemic-, and other heart disease). Primary renal diseases were grouped into seven categories: glomerulonephritis, adult polycystic kidney disease, diabetes mellitus, hypertension, pyelonephritis, unspecified kidney disease, and others kidney diseases. The employment status was defined as employed or not, according to the employment status the year before the patient started RRT. 

(iii) Statistical analysis

We described the frequency and distribution of patient characteristics in Table 1 based on treatment (transplantation and dialysis). We expressed continuous variables as mean and Standard Deviation (SD) and categorical variables as percentages. Between-group comparisons of continuous and categorical variables were done using t- and chi-square statistics, respectively. 

We used conventional logit models to study the association between SES and access to kidney transplantation, first in univariate models of the association between kidney transplantation and education and income respectively (model 1 in Table 2 and Table 3). For income, three step-wise multivariate models (models 2–4 in Table 2) were then estimated controlling for both income and education (model 2), demographic variables, i.e., age, gender, year of first RRT, marital status, home county, and citizenship (model 3) and clinical variables, i.e., primary renal disease and comorbidities (model 4). We included income and education simultaneously because education can be seen as a factor underlying the association between income and access to kidney transplantation. Education is generally defined early in life, and income is partly the result of educational achievements. Moreover, we can also explore which was the stronger independent factor which might inform inferences about mechanisms. For education, we did similar analysis without controlling for income in two step-wise multivariate models (models 2 and 3 in Table 3).

In order to assess if the association between treatment and socioeconomic factors are different depending on time in RRT we conducted a sensitivity analysis where the sample was divided based on pre-emptive or non-pre-emptive transplantation (patients received kidney transplantation before or after day 91 of RRT). For non-pre-emptive transplantation patients, we also used multivariate Cox proportional hazards regression analysis to assess the association between treatment and socioeconomic factors. We also conducted a sensitivity analysis where the sample were limited to the working aged patients (20–65 years of age) as there are concerns that employment status determines treatment choice [27]. 

Statistical significance was assumed for *p*-values < 0.05. All the statistical analyses were performed using Stata software, version 14.0 (College Station, TX, USA). The study has been approved by Lund Regional Ethical Review Board (Dnr: 2014/144).

## 3. Results

### 3.1. Baseline Characteristics

Patients were excluded if they had missing information for the main variables of interest, i.e., income (340 patients; 2.0%), education (525 patients; 3.1%), marital status (344 patients; 2.0%). We also excluded patients with negative income (22 patients; 0.1%) and extreme income (1 patient, 1.08 × 10^7^ SEK). The final sample constituted of 16,215 adult patients (age ≥ 18 years) with RRT, of whom 4392 (27.1%) received a living or deceased donor kidney transplantation by 31 December 2013. The frequency and distribution of patient baseline characteristics were displayed in Table 1. The mean age at start of RRT was 63.7 years old (SD: 15.1). Kidney transplantation patients were younger, healthier, higher educated, lived closer to a transplantation center, and had higher income compared to dialysis patients (*p* < 0.001). For example, 25% of the patients in the transplantation group had only mandatory education while this was the case for over 50% of patients in dialysis. A small but significant difference in terms of citizenship was also noted where, against expectation, the proportion with Swedish citizenship was higher in the dialysis group. 

### 3.2. Effects of SES on Access to Kidney Transplantation

For income, four logistic models were shown assessing the likelihood of receiving kidney transplantation (Table 2). The highest income group (quintile 5) had more than two times the likelihood of access to kidney transplantation compared with patients in the lowest group (quintile 1; reference group), as shown in the univariate model 1. The effect of high income decreased when simultaneously adjusting for education (model 2). Also adjusting for demographic variables (model 3) and clinical factors (model 4) increases the effect size and shows a clear positive association between income and likelihood of kidney transplantation. 

For education, three logistic models assessing the likelihood of receiving kidney transplantation were shown (Table 3). In the univariate analysis (model 1) patients with college education had more than five times greater chance of receiving kidney transplantation compared to patients with mandatory education. Although adjusting for other covariates reduced the effect of high education, higher education remains clearly positively associated with the likelihood of transplantation in the fully adjusted model (model 3).

Overall, the likelihood of access to kidney transplantation increased across both income and education. In both full models, a positive effect of younger age, being married, and having a Swedish citizenship was also noted. In the income model, neither gender nor living in the county of the transplantation center was shown to have any effect on access to kidney transplantation. However, in the education model, a positive effect of male gender was shown.

### 3.3. Sensitive Analyses

The sensitive analyses are presented in Table 4, Table 5, Table 6 and Table 7. Focusing on pre-emptive transplantations (model 1 in Table 4 and Table 5), the effect of high income and education is even higher than in the fully adjusted model which is also the case for the effect of citizenship. Interestingly, living in the county of a transplantation center now increased the likelihood of transplantation. Non-pre-emptive transplantation however (model 2 in Table 4 and Table 5) showed similar results as in the fully adjusted model, except that living in the county which has kidney transplantation center was now negatively associated with the chance of having non pre-emptive transplantation in the income model. Using Cox proportional hazards for non-pre-emptive transplantation patients, the results were similar as in model 2 in Table 4 and Table 5.

We assessed the probability of access to kidney transplantation for working aged patients which reduced the positive effect of income and education (model 3 in Table 4 and Table 5). Also, adjusting for employment status (model 4 in Table 4 and Table 5) substantially reduced the effects of income and education although remaining significantly associated. Being employed the year before start of RRT was strongly positively associated with the likelihood of receiving a kidney in both analysis for income and education. 

In the income models, no gender differences were found in any of the sensitivity analysis while younger age, being married, and a Swedish citizen remained positively associated with the likelihood of receiving kidney transplantation. The results of the sensitivity analysis of the education models were similar to those in the income models, except that male gender had a positive effect for non-pre-emptive transplantation patients (model 2 in Table 5) and working aged patients without adjustment for employment status (model 3 in Table 5).

## 4. Discussion

The results showed a strong association between SES and access to kidney transplantation. After multivariate analysis, patients in the most advantaged income quintile was associated with more than three times greater chance of receiving kidney transplantation compared with patients in the most disadvantaged quintile. An equally large effect was found for patients with higher education compared to patients with mandatory education only. 

The international Dialysis Outcomes and Practice Patterns Study (DOPPS) [18] found that income was highly positively associated with access to kidney transplantation although education was not when controlling for both variables, which is in contrast to our study. This difference may be due to DOPPS included patients from several countries, where education system are different and educational levels may have different meaning [14]. Patients in the DOPPS study were 18 to 65 years old, i.e., comparable with our sensitivity analysis on working aged patient which still showed a strong positive association between education and access to kidney transplantation.

Axelrod et al. [15] found that socioeconomic advantaged (mainly used post-codes as SES indices) were highly positively associated with both living- and deceased donor kidney transplantation while Grace et al. [9] found that socioeconomic advantaged were only highly positively associated with living-donor kidney transplantation, not with deceased-donor kidney transplantation. In order to compare with other studies, we also separated kidney transplantation to living- and deceased donor transplantation and our results were consistent with the study by Axelrod et al. [15] (results not shown). Schold et al. [16] found that higher income was associated with increased likelihood to receive a transplant, which was consistent with our results which did not separate living- and deceased donor kidney transplantation. Schaeffiner et al. [14] found higher educated patients had greater likelihood of access to kidney transplantation in the USA, which was consistent with our results. Thus, different studies using different definition of SES performed in different countries with different health care- and educational systems, all find that high socioeconomic status increases the chances of access to kidney transplantation.

Access to kidney transplantation probably depends on both patients and physicians. In Sweden, the decision of RRT was made by nephrologists, based on the Swedish guidelines [28] and the corresponding European guidelines [29]. In spite of this, in identifying potential transplant candidates, the possibility of physician bias cannot be ruled out [30,31]. However, it certainly is difficult to capture and therefore the reasons discussed below are only from the aspects of patients.

One suggested explanation for shown income and education discrepancies is communication barriers between patients and health care provider, which might be easier to overcome for higher SES patients [14]. This theory is possibly supported by our sensitive analysis results, where the effect size between income and education and access to pre-emptive transplantation (where human decision plays a great role) is substantially higher than that between income and education and later kidney transplantation. Another suggested explanation is that higher SES patients are better prepared for transplantation due to a known association between SES and medication and health advice compliance [32]. A third reason may be that higher SES patients actively seek living donors, while lower SES patients may be hindered in this due to a lack of awareness or means [10]. Moreover, there may be donor barriers to live donation in lower SES groups. Inferior health and high-risk behaviors are more common in lower SES population and social support networks may be poorer in these individuals. Therefore, prospective donors from lower SES populations may frequently be found medically unsuitable to donate a kidney [9]. 

Sweden is a high-income country with universal health care system with a goal to achieve equity in health. Sweden has relatively strong income equity and low degree of inequity in terms of education [33,34]. However, even in such a context SES are related to the likelihood of kidney transplantation. Other studies have found similar inequities in Sweden, for example related to drug utilization [35].

Interesting to note is that in the income models, there is no significant association between sex and access to kidney transplantation in our study, which is inconsistent with most other studies. This could potentially be explained by the large number of covariates included in the current study, including socioeconomic status. Further, our study confirmed the results of a previous study [11] where married patients had higher likelihood of kidney transplantation. The potential mechanism may be spousal kidney donation, more developed social networking, and more qualified for kidney transplantation duo to less depression [36] and positive health behaviors in terms of alcohol and drug abuse [37,38]. We also found that patients with Swedish citizenship had higher chance of access to kidney transplantation compared to non-Swedish patients, which was consistent with a French study [25]. The suggested reason may be that patients of non-Swedish were less likely to have an adequate Human Leukocyte Antigen (HLA)—and blood group-matched kidney than patients of European origin [25]. Socioeconomic and culture factors may also limit access to kidney transplantation [39], however Sweden provides healthcare coverage for other citizenship similar to Swedish citizenship so financial obstacles to kidney transplantation should be much smaller than in other countries. 

### Strengths and Limitations

We conducted multifaceted analyses of SES-related variables in relation to access to kidney transplantation by using individually linked national register data, which provided us vast and detailed information. As many confounding factors as possible were adjusted for in order to minimize systematic errors. Furthermore, the Swedish Renal Register has an excellent coverage which gives the study high power and excellent generalizability for end-stage renal disease patients. We used individual SES-related data, which is expected to give more accurate effect estimates compared to geographically defined SES. The measures of SES were also measured before start of RRT, thanks to the longitudinal nature of the data. We also took into account all major comorbidities before start of RRT which is better when controlling for confounding, compared to studies in USA [14], Australia [9] and Scotland [17]. Nonetheless, there are some potential limitations to our study. 

The present study was in absence of biochemical data (e.g., blood type, serum albumin level, levels of parathyroid hormone) which are known factors influencing access to kidney transplantation. However, these biochemical covariates are not expected to be strongly correlated with SES [40]. The outcome being defined as probability of access to kidney transplantation during the study period could influence the interpretation of inequity in access to kidney transplantation. Patients might die before having the chance to receive kidney transplantation, which is more likely to occur for the sicker and older patients. We have handled this risk by adjusting for age groups, primary renal disease, as well as comorbidities in the regression models. Moreover, using Cox proportional hazards for non-pre-emptive transplantation patients, the results were similar as what we got from logistic models. In addition, we could not identify factors influencing access to waiting list. However, studies have showed that for factors such as age and primary renal disease, differences persist at both stages. Finally, although we have controlled for important confounding factors, we lack information on other unobserved factors (e.g., other comorbidities, severity of comorbidities, adherence, race/ethnicity and physician bias). 

## 5. Conclusions

Access to kidney transplantation among Swedish end-stage renal disease patients is associated with common measures of socioeconomic status. Low income and education both reduced the chance of having kidney transplantation. However, reasons for these socioeconomic differences in the Swedish health care are unknown. The principal goal of Swedish healthcare system is to provide good and equal healthcare for all Swedish citizens. To this end, further studies are needed to identify the mechanisms of these inequities in order to construct effective interventions and policies. Moreover, since waiting list process is a key intermediate step to kidney transplantation, further studies can break down inequity of wait listing vs. inequity of kidney allocation, if data on the waiting list is available.

## Figures and Tables

**Table 1:** Baseline patient characteristics by treatment modality at the start of RRT.

Characteristics	Entire Study Population (*n* = 16,215)	Dialysis (*n* = 11,823)	Kidney Transplantation (*n* = 4392)	Pre-Emptive Tx (*n* = 612) ^#^	Living-Donor Tx (*n* = 1754)	Deceased-Do nor Tx (*n* = 2645)
Age (years) ***						
18–39	8.9	2.3	26.7	35.4	38.3	19.0
40–49	10.1	4.8	24.2	23.1	24.2	24.2
50–59	16.5	11.5	29.8	25.5	24.2	33.5
60+	64.6	81.4	19.4	16.0	13.3	23.4
**Male**	65.4	65.5	65.1	62.5	66.7	64.0
Year of first RRT ***	2004 (5.1)	2004 (5.2)	2003 (4.9)	2005 (5.3)	2004 (5.0)	2003 (4.8)
Education ***						
mandatory	46.2	54.1	25.1	15.4	19.7	28.6
high school	38.5	34.9	48.0	48.5	49.1	47.3
college	15.3	11.0	26.9	36.2	31.2	24.0
Marital status ***						
married	52.3	52.8	51.0	55.8	53.8	49.2
single	20.5	15.7	33.3	34.4	35.7	31.7
divorced	15.1	15.6	13.6	8.4	9.6	16.3
widow	12.1	15.9	2.1	1.5	0.9	2.8
Disposable income ***						
quintile 1 (0–99,998 SEK)	20.0	21.0	17.3	12.1	16.7	17.6
quintile 2 (100,002–122,743 SEK)	20.0	21.7	15.4	13.1	14.2	16.2
quintile 3 (122,755–146,224 SEK)	20.0	21.7	15.5	15.4	14.6	16.1
quintile 4 (146,231–188,732 SEK)	20.0	19.1	22.5	20.1	23.5	21.9
quintile 5 (188,751–6,685,735 SEK)	20.0	16.5	29.3	39.3	30.9	28.2
Swedish **	86.8	86.3	88.2	90.5	90.2	86.9
missing	9.6	10.5	7.4	6.1	5.6	8.5
Tx centers ***	48.8	47.9	51.1	57.1	51.9	50.6
Primary renal disease ***						
APKD	7.0	4.1	14.7	15.1	13.4	15.5
diabetic nephropathy	25.6	28.3	18.5	15.1	15.9	20.2
glomerulonephritis	15.3	10.0	29.5	31.2	34.4	26.2
hypertension	12.1	14.5	5.6	3.6	4.8	6.1
pyelonephritis	3.5	3.6	3.4	2.5	2.4	4.1
unspecified kidney disease	12.0	13.3	8.3	12.3	8.4	8.3
others	24.5	26.2	20.1	20.3	20.8	19.6
Comorbidities ***						
hypertension	70.4	71.0	68.8	67.3	68.8	68.7
diabetes mellitus	31.3	36.0	18.6	16.0	15.7	20.5
heart disease	35.0	43.8	11.2	6.6	9.0	12.6
cancer	12.6	16.2	3.0	2.5	2.9	3.0

RRT—renal replacement therapy; Tx—kidney transplantation; APKD—adult polycystic kidney disease; SEK—Swedish krona. Pre-emptive Tx can be either living- or deceased-donor Tx. Disposable income was divided into quintiles, where quintile 1 was the most disadvantaged and quintile 5 was the most advantaged. Continuous variables presented as mean (and standard deviations), categorical variables presented as percent of total. Groups (kidney transplantation vs. dialysis) were compared by *t*-test for continuous variables and by chi-square for categorical variables. ** *p* < 0.01; *** *p* < 0.001.

**Table 2:** The association between income and access to kidney transplantation by the multivariate logistic regression.

Variables Included in Model	Model 1 Crude OR of Income (95% CI)	Model 2 Adjusted for Education OR (95% CI)	Model 3 Adjusted for Model 2 + Demo. OR (95% CI)	Model 4 Adjusted for Model 3 + Clinical OR (95% CI)
**Disposable income (ref = quintile 1)**				
quintile 2 (100,002–122,743 SEK)	0.86 * (0.77–0.97)	0.80 *** (0.71–0.90)	1.02 (0.88–1.19)	1.12 (0.95–1.33)
quintile 3 (122,755–146,224 SEK)	0.87 * (0.78–0.98)	0.77 *** (0.68–0.87)	1.10 (0.94–1.28)	1.21 * (1.03–1.43)
quintile 4 (146,231–188,732 SEK)	1.44 *** (1.29–1.61)	1.10 (0.98–1.24)	1.79 *** (1.54–2.08)	1.91 *** (1.63–2.24)
quintile 5 (188,751–6,685,735 SEK)	2.16 *** (1.94–2.40)	1.40 *** (1.25–1.56)	3.23 *** (2.77–3.76)	3.22 *** (2.73–3.80)
**Education (ref = mandatory)**				
high school		2.80 *** (2.58–3.05)	1.46 *** (1.32–1.62)	1.48 *** (1.32–1.65)
college		4.56 *** (4.11–5.07)	2.47 *** (2.16–2.82)	2.35 *** (2.03–2.72)
**Age at referral (years) (ref = 18 to 39)**				
40 to 49			0.36 *** (0.30–0.43)	0.36 *** (0.30–0.44)
50 to 59			0.15 *** (0.13–0.18)	0.17 *** (0.14–0.21)
60+			0.02 *** (0.01–0.02)	0.02 *** (0.02–0.03)
**Sex (ref = female)**				
male			0.91 (0.83–1.01)	0.99 (0.89–1.10)
**Year of first RRT**			0.94 *** (0.93–0.95)	0.94 *** (0.93–0.95)
**Marital status (ref = married)**				
single			0.59 *** (0.52–0.66)	0.57 *** (0.50–0.66)
divorced			0.63 *** (0.55–0.72)	0.65 *** (0.56–0.75)
widow			0.31 *** (0.24–0.39)	0.29 *** (0.23–0.37)
**Citizenship (ref = non-Swedish)**				
Swedish			1.36 ** (1.08–1.72)	1.43 ** (1.11–1.84)
missing			0.58 *** (0.44–0.78)	0.59 ** (0.43–0.80)
**Home county (ref = non Tx centers)**				
Tx centers			0.94 (0.86–1.03)	0.92 (0.83–1.01)
**Primary renal disease (ref = APKD)**				
diabetic nephropathy				0.26 *** (0.21–0.33)
glomerulonephritis				0.76 ** (0.62–0.92)
hypertension				0.29 *** (0.23–0.37)
pyelonephritis				0.52 *** (0.38–0.69)
unspecified kidney disease				0.29 *** (0.23–0.36)
others				0.32 *** (0.27–0.39)
**Comorbidities (ref = non comorbidities)**				
hypertension				1.74 *** (1.52–1.99)
diabetes mellitus				0.57 *** (0.48–0.67)
heart disease				0.29 *** (0.26–0.33)
cancer				0.23 *** (0.18–0.28)

ref—reference group; OR—odds ratio; CI—confidence interval. Model 1: Crude OR of disposable income; Model 2: ORs were adjusted for education; Model 3: model 2 + demographic variables (age at referral, sex, year of first RRT, marital status, citizenship, and home county); Model 4: model 3 + clinical variables, which included primary renal disease and comorbidities. *** *p* < 0.001; ** *p* < 0.01; * *p* < 0.05.

**Table 3:** The association between education and access to kidney transplantation by the multivariate logistic regression.

Variables Included in Model	Model 1 Crude OR of Education (95% CI)	Model 2 Adjusted for Demo. OR (95% CI)	Model 3 Adjusted for Model 2 + Clinical OR (95% CI)
**Education (ref = mandatory)**			
high school	2.97 *** (2.73–3.23)	1.67 *** (1.51–1.85)	1.68 *** (1.51–1.88)
college	5.25 *** (4.74–5.81)	3.36 *** (2.96–3.82)	3.18 *** (2.77–3.66)
**Age at referral (years) (ref = 18 to 39)**			
40 to 49		0.39 *** (0.33–0.46)	0.40 *** (0.33–0.48)
50 to 59		0.19 *** (0.16–0.22)	0.21 *** (0.18–0.26)
60+		0.02 *** (0.01–0.02)	0.02 *** (0.02–0.03)
**Sex (ref = female)**			
male		1.06 (0.96–1.16)	1.14 * (1.03–1.26)
**Year of first RRT**		0.96 *** (0.95–0.97)	0.96 *** (0.95–0.97)
**Marital status (ref = married)**			
single		0.57 *** (0.51–0.65)	0.56 *** (0.50–0.64)
divorced		0.63 *** (0.55–0.72)	0.65 *** (0.57–0.75)
widow		0.34 *** (0.27–0.43)	0.32 *** (0.25–0.41)
**Citizenship (ref = non-Swedish)**			
Swedish		1.64 *** (1.31–2.06)	1.73 *** (1.35–2.22)
missing		0.74 * (0.56–0.98)	0.72 * (0.53–0.98)
**Home county (ref = non Tx centers)**			
Tx centers		0.99 (0.91–1.08)	0.96 (0.87–1.06)
**Primary renal disease (ref = APKD)**			
diabetic nephropathy			0.27 *** (0.21–0.34)
glomerulonephritis			0.77 ** (0.64–0.94)
hypertension			0.28 *** (0.22–0.35)
pyelonephritis			0.50 *** (0.38–0.67)
unspecified kidney disease			0.28 *** (0.23–0.35)
others			0.32 *** (0.27–0.38)
**Comorbidities (ref = non comorbidities)**			
hypertension			1.73 *** (1.52–1.97)
diabetes mellitus			0.54 *** (0.46–0.64)
heart disease			0.29 *** (0.26–0.33)
cancer			0.23 *** (0.19–0.29)

Model 1: Crude OR of education; Model 2: ORs were adjusted for demographic variables (age at referral, sex, year of first RRT, marital status, citizenship, and home county); Model 3: model 2 + clinical variables, which included primary renal disease and comorbidities. *** *p* < 0.001; ** *p* < 0.01; * *p* < 0.05.

**Table 4:** Sensitive analyses for income by the multivariate logistic regression.

Variables Included in Model	Model 1 for Pre-Emptive Transplantation Sample (*n* = 12,624)	Model 2 for Later Kidney Transplantation Sample (*n* = 15,414)	Model 3 for Working Aged Patients Sample (*n* = 6844)	Model 4 for Working Aged Patients Sample + Employment Status (*n* = 6844)
**Disposable income (ref = quintile 1)**				
quintile 2 (100,002–122,743 SEK)	1.19 (0.84–1.69)	1.11 (0.94–1.32)	1.09 (0.90–1.33)	1.04 (0.85–1.27)
quintile 3 (122,755–146,224 SEK)	1.63 ** (1.16–2.28)	1.15 (0.97–1.37)	1.07 (0.88–1.30)	0.96 (0.79–1.17)
quintile 4 (146,231–188,732 SEK)	2.32 *** (1.67–3.22)	1.86 *** (1.57–2.20)	1.64 *** (1.35–1.98)	1.30 ** (1.06–1.58)
quintile 5 (188,751–6,685,735 SEK)	4.00 *** (2.89–5.53)	3.06 *** (2.58–3.64)	2.50 *** (2.05–3.05)	1.69 *** (1.37–2.08)
**Education (ref = mandatory)**				
high school	1.94 *** (1.53–2.48)	1.44 *** (1.28–1.62)	1.39 *** (1.22–1.59)	1.32 *** (1.15–1.51)
college	3.50 *** (2.64–4.64)	2.23 *** (1.92–2.60)	2.32 *** (1.94–2.77)	2.00 *** (1.66–2.39)
**Age at referral (years) (ref = 18 to 39)**				
40 to 49	0.22 *** (0.16–0.30)	0.38 *** (0.31–0.47)	0.40 *** (0.33–0.49)	0.38 *** (0.31–0.47)
50 to 59	0.09 *** (0.07–0.13)	0.19 *** (0.15–0.23)	0.18 *** (0.15–0.22)	0.19 *** (0.16–0.23)
60 +	0.01 *** (0.01–0.02)	0.02 *** (0.02–0.03)	0.08 *** (0.07–0.10)	0.10 *** (0.08–0.13)
**Sex (ref = female)**				
male	0.88 (0.72–1.08)	1.03 (0.92–1.16)	1.07 (0.94–1.21)	1.05 (0.92–1.19)
**Year of first RRT**	1.02 (1.00–1.04)	0.92 *** (0.91–0.93)	0.92 *** (0.91–0.93)	0.92 *** (0.90–0.93)
**Marital status (ref = married)**				
single	0.38 *** (0.30–0.49)	0.62 *** (0.54–0.71)	0.49 *** (0.43–0.57)	0.53 *** (0.45–0.61)
divorced	0.33 *** (0.24–0.46)	0.70 *** (0.60–0.81)	0.52 *** (0.44–0.61)	0.56 *** (0.48–0.66)
widow	0.21 *** (0.11–0.40)	0.31 *** (0.24–0.40)	0.43 *** (0.29–0.64)	0.50 *** (0.33–0.74)
**Citizenship (ref = non-Swedish)**				
Swedish	2.46 ** (1.47–4.11)	1.41 ** (1.09–1.83)	1.60 *** (1.23–2.10)	1.41 * (1.08–1.85)
missing	1.22 (0.65–2.30)	0.54 *** (0.40–0.75)	0.77 (0.55–1.10)	0.60 ** (0.42–0.85)
**Home county (ref = non Tx centers)**				
Tx centers	1.22 * (1.008–1.48)	0.88 * (0.80–0.98)	0.95 (0.84–1.07)	0.94 (0.84–1.06)
**Primary renal disease (ref = APKD)**				
diabetic nephropathy	0.25 *** (0.17–0.38)	0.26 *** (0.21–0.33)	0.27 *** (0.21–0.35)	0.28 *** (0.21–0.37)
glomerulonephritis	0.68 * (0.49–0.94)	0.78 * (0.63–0.95)	0.78 * (0.62–0.99)	0.77 * (0.60–0.98)
hypertension	0.21 *** (0.12–0.34)	0.31 *** (0.24–0.39)	0.40 *** (0.30–0.55)	0.43 *** (0.31–0.58)
pyelonephritis	0.39 ** (0.22–0.72)	0.55 *** (0.41–0.75)	0.79 (0.53–1.17)	0.78 (0.53–1.16)
unspecified kidney disease	0.32 *** (0.22–0.46)	0.28 *** (0.22–0.35)	0.37 *** (0.28–0.49)	0.39 *** (0.29–0.51)
others	0.28 *** (0.20–0.39)	0.34 *** (0.28–0.41)	0.38 *** (0.31–0.48)	0.40 *** (0.32–0.50)
**Comorbidities (ref = non comorbidities)**				
hypertension	1.16 (0.91–1.47)	1.89 *** (1.64–2.17)	1.70 *** (1.45–1.99)	1.63 *** (1.39–1.92)
diabetes mellitus	0.49 *** (0.35–0.69)	0.58 *** (0.49–0.70)	0.60 *** (0.49–0.74)	0.66 *** (0.54–0.81)
heart disease	0.20 *** (0.15–0.28)	0.31 *** (0.27–0.36)	0.35 *** (0.30–0.41)	0.38 *** (0.32–0.44)
cancer	0.14 *** (0.08–0.24)	0.24 *** (0.19–0.30)	0.25 *** (0.19–0.33)	0.25 *** (0.19–0.34)
**Employment (ref = non-employment)**				
employment				2.55 *** (2.23–2.92)

*** *p* < 0.001; ** *p* < 0.01; * *p* < 0.05.

**Table 5:** Sensitive analyses for education by the multivariate logistic regression.

Variables Included in Model	Model 1 for Pre-Emptive Transplantation Sample (*n* = 12,624)	Model 2 for Later Kidney Transplantation Sample (*n* = 15,414)	Model 3 for Working Aged Patients Sample (*n* = 6844)	Model 4 for Working Aged Patients Sample + Employment Status (*n* = 6844)
**Education (ref = mandatory)**				
high school	2.27 *** (1.79–2.87)	1.64 *** (1.46–1.84)	1.51 *** (1.33–1.72)	1.36 *** (1.19–1.56)
college	4.89 *** (3.73–6.41)	3.01 *** (2.60–3.49)	2.87 *** (2.41–3.41)	2.19 *** (1.83–2.62)
**Age at referral (years) (ref = 18 to 39)**				
40 to 49	0.25 *** (0.18–0.33)	0.42 *** (0.35–0.51)	0.43 *** (0.35–0.52)	0.39 *** (0.32–0.48)
50 to 59	0.13 *** (0.09–0.17)	0.23 *** (0.19–0.28)	0.21 *** (0.17–0.26)	0.21 *** (0.17–0.25)
60+	0.01 *** (0.01–0.02)	0.03 *** (0.02–0.03)	0.10 *** (0.08–0.12)	0.12 *** (0.09–0.14)
**Sex (ref = female)**				
male	1.06 (0.87–1.29)	1.17 ** (1.05–1.31)	1.19 ** (1.05–1.35)	1.11 (0.98–1.26)
**Year of first RRT**	1.04 ** (1.02–1.06)	0.94 *** (0.93–0.95)	0.93 *** (0.92–0.95)	0.92 *** (0.91–0.94)
**Marital status (ref = married)**				
single	0.38 *** (0.30–0.49)	0.61 *** (0.53–0.70)	0.49 *** (0.42–0.56)	0.52 *** (0.45–0.61)
divorced	0.33 *** (0.24–0.46)	0.71 *** (0.61–0.82)	0.51 *** (0.44–0.60)	0.57 *** (0.48–0.67)
widow	0.23 *** (0.12–0.45)	0.34 *** (0.26–0.44)	0.51 *** (0.34–0.75)	0.55 *** (0.37–0.82)
**Citizenship (ref = non-Swedish)**				
Swedish	3.40 *** (2.04–5.65)	1.68 *** (1.30–2.17)	1.88 *** (1.45–2.45)	1.51 ** (1.16–1.97)
missing	1.66 (0.89–3.12)	0.67 * (0.49–0.91)	0.92 (0.65–1.29)	0.63 ** (0.44–0.90)
**Home county (ref = non Tx centers)**				
Tx centers	1.31 ** (1.09–1.58)	0.92 (0.83–1.02)	0.98 (0.87–1.10)	0.96 (0.85–1.08)
**Primary renal disease (ref = APKD)**				
diabetic nephropathy	0.27 *** (0.18–0.40)	0.27 *** (0.21–0.34)	0.27 *** (0.21–0.36)	0.28 *** (0.21–0.37)
glomerulonephritis	0.73 (0.53–1.00)	0.79 * (0.65–0.97)	0.79 * (0.62–0.99)	0.77 * (0.61–0.98)
hypertension	0.20 *** (0.12–0.33)	0.30 *** (0.23–0.37)	0.38 *** (0.28–0.51)	0.41 *** (0.30–0.56)
pyelonephritis	0.39 ** (0.22–0.72)	0.54 *** (0.40–0.73)	0.77 (0.52–1.14)	0.78 (0.52–1.15)
unspecified kidney disease	0.33 *** (0.22–0.47)	0.27 *** (0.21–0.34)	0.36 *** (0.28–0.47)	0.38 *** (0.29–0.51)
others	0.29 *** (0.21–0.40)	0.34 *** (0.28–0.41)	0.38 *** (0.30–0.48)	0.40 *** (0.32–0.50)
**Comorbidities (ref= non comorbidities)**				
hypertension	1.13 (0.89–1.43)	1.88 *** (1.64–2.16)	1.70 *** (1.45–1.99)	1.62 *** (1.38–1.91)
diabetes mellitus	0.46 *** (0.33–0.64)	0.56 *** (0.47–0.67)	0.58 *** (0.48–0.71)	0.66 *** (0.54–0.81)
heart disease	0.21 *** (0.15–0.28)	0.31 *** (0.27–0.36)	0.35 *** (0.30–0.41)	0.38 *** (0.32–0.44)
cancer	0.15 *** (0.09–0.25)	0.25 *** (0.20–0.31)	0.25 *** (0.19–0.33)	0.26 *** (0.19–0.34)
**Employment (ref = non-employment)**				
employment				2.90 *** (2.55–3.29)

*** *p* < 0.001; ** *p* < 0.01; * *p* < 0.05.

**Table 6:** Sensitive analyses of the association between income and access to non-pre-emptive transplantation by Cox proportional hazards model (*n* = 15,414).

Variables Included in Model	Model 1 Crude HR of Income (95% CI)	Model 2 Adjusted for Education HR (95% CI)	Model 3 Adjusted for Model 2 + Demo. HR (95% CI)	Model 4 ADJUSTED for Model 3 + Clinical HR (95% CI)
**Disposable income (ref = quintile 1)**				
quintile 2 (100,002–122,743 SEK)	0.88 * (0.78–0.98)	0.84 ** (0.75–0.94)	1.03 (0.92–1.16)	1.08 (0.96–1.21)
quintile 3 (122,755–146,224 SEK)	0.85** (0.76–0.96)	0.78 *** (0.70–0.88)	1.00 (0.89–1.12)	1.07 (0.95–1.20)
quintile 4 (146,231–188,732 SEK)	1.33 *** (1.20–1.47)	1.08 (0.97–1.20)	1.47 *** (1.32–1.63)	1.50 *** (1.35–1.67)
quintile 5 (188,751–6,685,735 SEK)	1.73 *** (1.56–1.91)	1.23 *** (1.11–1.36)	1.98 *** (1.78–2.20)	1.93 *** (1.73–2.15)
**Education (ref = mandatory)**				
high school		2.18 *** (2.02–2.37)	1.26 *** (1.16–1.37)	1.23 *** (1.14–1.34)
college		3.10 *** (2.82–3.41)	1.85 *** (1.68–2.03)	1.71 *** (1.55–1.88)
**Age at referral (years) (ref = 18 to 39)**				
40 to 49			0.60 *** (0.55–0.66)	0.62 *** (0.56–0.68)
50 to 59			0.36 *** (0.33–0.40)	0.40 *** (0.36–0.44)
60+			0.07 *** (0.06–0.08)	0.09 *** (0.08–0.10)
**Sex (ref = female)**				
male			1.03 (0.96–1.11)	1.05 (0.98–1.13)
**Year of first RRT**			0.98 *** (0.97–0.98)	0.99 *** (0.98–0.99)
**Marital status (ref = married)**				
single			0.79 *** (0.73–0.86)	0.80 *** (0.74–0.87)
divorced			0.78 *** (0.71–0.86)	0.80 *** (0.73–0.88)
widow			0.39 *** (0.31–0.49)	0.39 *** (0.31–0.49)
**Citizenship (ref = non-Swedish)**				
Swedish			1.33 *** (1.14–1.57)	1.44 *** (1.22–1.69)
missing			1.18 (0.96–1.44)	1.25 * (1.01–1.53)
**Home county (ref = non Tx centers)**				
Tx centers			0.93 * (0.87–0.99)	0.91 ** (0.86–0.98)
**Primary renal disease (ref = APKD)**				
diabetic nephropathy				0.56 *** (0.48–0.65)
glomerulonephritis				0.87 * (0.78–0.97)
hypertension				0.52 *** (0.44–0.61)
pyelonephritis				0.70 *** (0.57–0.85)
unspecified kidney disease				0.52 *** (0.45–0.61)
others				0.57 *** (0.51–0.64)
**Comorbidities (ref = non comorbidities)**				
hypertension				1.25 *** (1.15–1.36)
diabetes mellitus				0.66 *** (0.58–0.75)
heart disease				0.46 *** (0.41–0.51)
cancer				0.42 *** (0.35–0.51)

HR—hazard ratio. Model 1: Crude HR of disposable income; Model 2: HRs were adjusted for education; Model 3: model 2 + demographic variables (age at referral, sex, year of first RRT, marital status, citizenship, and home county); Model 4: model 3 + clinical variables, which included primary renal disease and comorbidities. *** *p* < 0.001; ** *p* < 0.01; * *p* < 0.05.

**Table 7:** Sensitive analyses of the association between education and access to non-pre-emptive transplantation by Cox proportional hazards model (*n* = 15,414).

Variables Included in Model	Model 1 Crude HR of Education (95% CI)	Model 2 Adjusted for Demo. HR (95% CI)	Model 3 Adjusted for Model 2 + Clinical HR (95% CI)
**Education (ref = mandatory)**			
high school	2.28 *** (2.11–2.47)	1.39 *** (1.28–1.51)	1.36 *** (1.25–1.47)
college	3.46 *** (3.16–3.78)	2.22 *** (2.02–2.44)	2.02 *** (1.84–2.22)
**Age at referral (years) (ref = 18 to 39)**			
40 to 49		0.64 *** (0.58–0.70)	0.65 *** (0.59–0.71)
50 to 59		0.41 *** (0.37–0.45)	0.45 *** (0.41–0.50)
60+		0.08 *** (0.07–0.09)	0.10 *** (0.09–0.12)
**Sex (ref = female)**			
male		1.12 ** (1.04–1.20)	1.13 ** (1.05–1.21)
**Year of first RRT**		0.99 *** (0.98–0.99)	0.99 (0.99–1.00)
**Marital status (ref = married)**			
single		0.80 *** (0.73–0.86)	0.81 *** (0.75–0.88)
divorced		0.78 *** (0.71–0.86)	0.81 *** (0.73–0.89)
widow		0.41 *** (0.33–0.52)	0.41 *** (0.33–0.52)
**Citizenship (ref = non-Swedish)**			
Swedish		1.52 *** (1.30–1.78)	1.63 *** (1.39–1.91)
missing		1.35 ** (1.10–1.65)	1.42 ** (1.15–1.74)
**Home county (ref = non Tx centers)**			
Tx centers		0.95 (0.89–1.02)	0.94 (0.88–1.00)
**Primary renal disease (ref = APKD)**			
diabetic nephropathy			0.56 *** (0.48–0.65)
glomerulonephritis			0.89 * (0.80–0.99)
hypertension			0.51 *** (0.44–0.60)
pyelonephritis			0.70 *** (0.57–0.85)
unspecified kidney disease			0.51 *** (0.44–0.60)
others			0.57 *** (0.51–0.64)
**Comorbidities (ref = non comorbidities)**			
hypertension			1.25 *** (1.14–1.36)
diabetes mellitus			0.65 *** (0.57–0.74)
heart disease			0.45 *** (0.41–0.51)
cancer			0.42 *** (0.35–0.51)

Model 1: Crude HR of education; Model 2: HRs were adjusted for demographic variables (age at referral, sex, year of first RRT, marital status, citizenship, and home county); Model 3: model 2 + clinical variables, which included primary renal disease and comorbidities. *** *p* < 0.001; ** *p* <0.01; * *p* < 0.05.
